# A scoping review of the methods used to estimate health facility catchment populations for child health indicators in sub-Saharan Africa

**DOI:** 10.1186/s12963-025-00374-0

**Published:** 2025-03-29

**Authors:** Matthew Johnson, Wole Ademola Adewole, Victor Alegana, C. Edson Utazi, Nuala McGrath, James Wright

**Affiliations:** 1 School of Geography and Environmental Science, University of Southampton, Building 44, University Road, Southampton, SO17 1BJ UK; 2 Kenya Medical Research Institute, Wellcome Trust Research Programme, Nairobi, Kenya; 3 WorldPop, School of Geography and Environmental Science, University of Southampton, Southampton, UK; 4 Department of Social Statistics and Demography, Faculty of Social, Human and Mathematical Sciences, University of Southampton, Southampton, UK; 5 School of Primary Care, Population Sciences and Medical Education, Faculty of Medicine, University of Southampton, Southampton, UK; 6 Africa Health Research Institute, Durban, KwaZulu-Natal South Africa; 7 School of Nursing and Public Health, University of KwaZulu-Natal, Durban, South Africa

**Keywords:** Health facility, Catchment area, Denominator, Population, Demography, Spatial, Child health, Sub-Saharan Africa, Scoping review

## Abstract

**Background:**

Evidence indicating persistent geographic inequalities in health outcomes signifies a need for routine subnational monitoring of health-related Sustainable Development Goal targets in sub-Saharan Africa. Health facilities may be an appropriate subnational unit for monitoring purposes, but a lack of suitable demographic data complicates the production of baseline facility-level population denominators against which progress can be reliably measured. This scoping review aimed to map the methods and data sources used to estimate health facility catchment areas and translate them to population denominators for child health indicators in the region.

**Methods:**

Peer-reviewed research publications and grey literature reports were identified by searching bibliographic databases and relevant organisational websites. The inclusion criteria required that studies were conducted in sub-Saharan Africa since January 2000, described quantitative method(s) for estimating health facility catchment areas and/or population denominators, and focussed on children as the population of interest. Following title/abstract then full text screening of search results, relevant data were extracted using a standard form. Thematic analysis was undertaken to extract themes and present a narrative synthesis.

**Results:**

Overall, 33 research publications and 3 grey literature reports were included. Of these, only 7 research studies and 1 technical guidance document outlined aims explicitly framed around methods development and/or evaluation. Studies increasingly estimated catchment areas using complex geostatistical or travel time-based modelling approaches rather than simpler proximity metrics, and produced denominators by intersecting catchment boundaries with gridded population surfaces rather than aggregating area-based administrative counts. Few studies used data produced by or describing health facilities to link estimation methods to service utilisation patterns, inter-facility competition or facility characteristics.

**Conclusion:**

There is a need for catchment population estimation methods that can be scaled to national-level facility networks and replicated across the region. This could be achieved by leveraging routinely collected health data and other readily available and nationally consistent data sources. Future methodological development should emphasise modern geostatistical approaches drawing upon the relative strengths of multiple data sources and capturing the range of spatial, supply-side, individual-level and environmental factors with potential to influence catchments’ extent, shape and demographic composition.

**Supplementary Information:**

The online version contains supplementary material available at 10.1186/s12963-025-00374-0.

## Background

Projections from the 2017 Global Burden of Disease study suggest that many countries of sub-Saharan Africa (SSA) are falling short of the progress required to meet any health-related Sustainable Development Goals (SDGs) target by 2030 [1]. The region also faces challenges in relation to child health; despite recent improvement, levels of mortality [2, 3] and infectious disease incidence [4, 5] remain high, amid evidence of growing non-communicable disease burden [6]. Subnational analyses, however, reveal within-country inequalities in the distribution of health outcomes [7–9] that would otherwise be hidden by national-level data, signifying a need for routine monitoring at more granular geographies. By revealing and characterising high risk areas or underserved populations such an approach could also help to address spatial inequalities, contributing to targeted resource allocation and the development of locally-relevant interventions and services [1, 2, 7].

Health facilities (HFs) may be an appropriate subnational unit for monitoring progress against targets in SSA: they provide routine, formal care to populations in small geographic areas and, in so doing, collect continuous and near real-time empirical data describing service utilisation, health status, disease incidence and prevalence, and intervention coverage [10, 11]. Intuitively, progress monitoring at this level presupposes clear knowledge of the geographical ‘catchment’ area served by each HF, together with its baseline denominator population and demographic composition [12]. Though traditionally viewed as the principal sources of demographic data in many low- and middle-income countries (LMICs), censuses and household surveys do not provide direct population estimates at the lowest levels of health service delivery [13]. Moreover, as catchments are rarely delineated by unambiguous administrative boundaries in SSA, so-called ‘natural’ catchments predominate, tending to emerge as a product of interacting factors influencing patient choice [14], including HF type [15], service quality [16, 17] or distance decay (meaning the tendency towards waning utilisation with greater travel distance) [18, 19]. Without the benefit of typical demographic data and methods, a range of statistical and geospatial model-based approaches to the estimation of HF catchment areas and population denominators have been developed, many of which account for these, and other, salient factors [20], but seldom incorporate the data collected by HFs themselves as the product of routine patient care.

Nonetheless, the view that routinely collected health data (RCHD) could be better leveraged for population health improvement has gained traction in recent years, with renewed efforts to improve their quality [21, 22] and establish them as a source of intelligence to monitor health indicators and inform local, evidence-based decision-making [23–25]. Meanwhile, District Health Information Software (DHIS2), a health management information system (HMIS) for the collection, warehousing and reporting of RCHD, has been adopted by more than 70 LMICs covering some 30% of the world’s population, principally in SSA and south/south east Asia [26], thus strengthening and harmonising their data collection and production infrastructure. These developments are emblematic of the rapidly evolving data landscape of SSA and may have precipitated methodological innovation that could be replicated more widely across the region. This scoping review was conducted with the aims of mapping the: (i) methods and data sources that have been used to estimate HF catchment areas and translate them to population denominators for child health indicators in SSA; (ii) approaches used to evaluate these estimation methods.

## Methods

The review followed the methodological framework established by Arksey and O’Malley [27], and is reported in accordance with the Preferred Reporting Items for Systematic reviews and Meta-Analyses Extension for Scoping Reviews (PRISMA-ScR) checklist [28] (Supplementary file 1). A protocol was also registered on Open Science Framework [29].

### Identifying and selecting peer-reviewed publications

Publications were identified using a search strategy developed by the study team and reviewed by two research librarians. Database search strings (Supplementary file 2) consisted of MeSH terms (Medline only) and search terms arranged into four broad ‘concepts’ (children, HFs, catchment areas/population denominators and SSA) using Boolean operators. Exploratory scoping searches were used to determine the combination of terms required to capture each ‘concept’. Searches of Medline, Scopus, Web of Science Core Collection, GeoBase and African Index Medicus bibliographic databases were executed on 25th October 2021. Results were imported into an EndNote X9 (Clarivate Analytics, Philadelphia, USA) database and de-duplicated.

Screening against the inclusion criteria (Table 1) was conducted in two stages: (i) title/abstract screening of de-duplicated search results using the R ‘metagear’ package [30]; (ii) full text screening of results passing the first stage. Where publications were unavailable online, the authors were contacted directly. Prior to each stage a random sample of 20% were independently screened by MJ and WAA to calibrate the inclusion criteria and screening approach [28]. Discrepancies between reviewers were discussed and, where necessary, resolved by JW as arbitrator. Agreement was assessed using Cohen’s kappa coefficient; once a minimum value of 0.80 was achieved, all remaining publications were screened by MJ alone [31]. After completion, the list of full text screening decisions and rationale was verified by WAA and JW. Reference lists were searched to identify additional publications meeting the inclusion criteria.

**Table 1:** Inclusion criteria used to select peer-reviewed publications for the review

Criterion	Description	Additional notes
1	Publication written in English or French languages	• Database searches were conducted in English
2	Publication dates from January 2000 onwards	• Selected as efforts to strengthen HMIS [21] and uptake of DHIS2 [32] gathered pace during the 2000s
3	Publication describes a study conducted in at least one country from the United Nations SDG SSA regional grouping [33]	
4	Publication describes a quantitative method(s) for estimating catchment areas and/or population denominators	• Publications describing the development of a novel method or implementation of an existing method were considered relevant, but methods must involve estimation or modelling as opposed to delineating or visualising empirically observed health-seeking flows • Publications describing the application of similar methods as an intermediate step to testing associations between health service accessibility and outcomes were also considered relevant to the review
5	The method(s) associates catchment areas and/or population denominators with specific health facilities as the unit of analysis	• ‘Specific’ may mean the nearest or named health facilities
6	Children of any age are the/a specific population subgroup of interest	• Many health-related SDG indicators are targeted to a demographic subgroup as opposed to the total population [34] • Several countries of SSA have expanded the set of child health services covered by free healthcare policies in recent years which, though the evidence is mixed, may have resulted in increased service utilisation and consequential growth in the volume of routine data [35]

### Grey literature

Recognising non-academic organisations’ role in the production of methodological and technical guidance, grey literature searches were executed during January, February and June 2022. Google Scholar and relevant organisational websites were searched using a simplified strategy consisting of keyword combinations representing ‘concepts’ used to identify peer-reviewed publications (Supplementary file 3). As grey literature tends to appear more regularly after around 30 pages of Google Scholar search results [36], the first 50 pages (500 results) were screened by title/preview only, as were the results from organisational websites. EBSCO was searched as a general source of grey literature and ProQuest for dissertations and theses. Results from peer-reviewed journals were excluded. Title/abstract screening used the platforms’ web interfaces, with results passing this stage progressing to full text screening. Screening was conducted by MJ alone and, other than relaxing the third criterion (Table 1) to allow inclusion of methodological guidance not linked to specific countries, followed the approach used for peer-reviewed publications.

### Data extraction and synthesis

For consistency, a standard form was developed to extract variables including bibliographic information, study setting and population, data sources, software and methods used, results, findings and limitations. Following independent extraction from 15% of peer-reviewed publications by MJ and WAA as a calibration exercise [28], the remainder were reviewed by MJ alone. After completion, the extracted data were verified by WAA. R v4.0.2 and RStudio v1.3.1073 (R Core Team, Vienna, Austria) were used to create quantitative tables summarising the corpus of publications. Thematic analysis was undertaken to extract key themes and present a narrative synthesis.

## Results

**Fig. 1:**
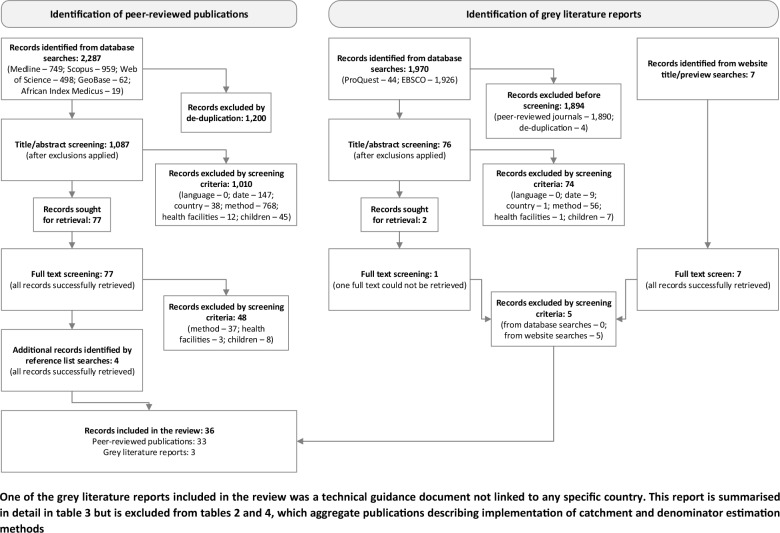
PRISMA flow diagram outlining the process used to identify and select relevant publications and reports

A PRISMA flow diagram outlining the selection process is presented in Fig. 1. Twenty-nine of 1087 unique peer-reviewed publications were included, as well as 4 identified via reference list searches. Also included were 3 grey literature reports, consisting of one case study [37], one thesis [38] and one technical guidance document [39], of which the latter is excluded from the forthcoming quantitative tables and synthesis.

### Study setting and health facility locations

Excepting 2 region-wide [40, 41] and 2 multi-country studies [37, 42], most (88.6%) peer-reviewed publications and grey literature reports (hereafter, publications) described single-country studies (Table 2). Overall, 15 countries were represented; while most had 3 publications or fewer, 13 (37.1%) originated from Kenya. Although 7 (20.0%) publications implemented methods across the national HF network [43–49], most (68.6%) analysed a defined subnational area or subset of specific, purposively selected HFs. Only 3 (8.6%) publications stated that analyses captured public and private sector HFs [42, 46, 50] (Table 3). All studies utilised data describing HF locations (Table 4). Although study staff sometimes (14.3%) conducted onsite geolocation by field survey [15, 42, 44, 51, 52], information held within the national health system or by HFs were the most common data source (60.0%). Recent years have seen the development of open databases geolocating public sector HFs across SSA [53, 54], and there were instances of their use to augment within-country data [46], or as the primary source for region-wide analyses [40, 41].

**Table 2:** Geographic area and scale of the included publications

Area	Regional	National	Subnational^a^	Single health facility	Number of publications^b^
Sub-Saharan Africa region	2				2
Multi-country study			2		2
Burkina Faso				1	1
Democratic Republic of the Congo			1		1
Ghana			1		1
Kenya		1	11	1	13
Madagascar			1		1
Malawi		1			1
Mozambique		1	1		2
Namibia			1		1
Niger		1			1
Rwanda			1		1
Somaliland		1			1
South Africa		1			1
Tanzania			1	1	2
The Gambia			1		1
Uganda		1	2		3
Total (%)	2 (5.7)	7 (20.0)	23 (65.7)	3 (8.6)	35 (100.0)

^a^ ‘Subnational’ denotes a defined subnational area or a subset of specific, purposively selected health facilities

^b^Table includes 33 peer-reviewed publications and 2 (of 3) grey literature reports (1 technical guidance document has been excluded)

**Table 3:** Detailed summary of the data extracted from the publications included in the review

References	Country of origin	Scale	Product	Population	Health indicator	Health facilities analysed	Software	Estimation methods	Evaluation methods	Relevant estimation/evaluation outputs
Noor et al. [52]	Kenya	Subnational	Methods development and/or evaluation	Paediatric patients	Malaria/fever treatment seeking	174 government hospitals, health centres and dispensaries	ArcView	• Catchment areas estimated using Thiessen polygons (straight-line distance) and the point-in-polygon method to encapsulate enumeration area centroids within each polygon • Catchment denominator estimated as the total census population for all included enumeration areas	Onsite survey of service users to collect origin/destination data for statistical comparison of estimated catchment areas and empirical utilisation patterns	Maps separately visualising catchment areas post-estimation and based on actual utilisation; distance decay curve for indicator
Gething et al. [51]	Kenya	Subnational	Methods development and/or evaluation	Paediatric patients	Febrile outpatient attendance	81 government hospitals, health centres and dispensaries	ArcView	• Preliminary catchment areas produced using Thiessen polygons (straight-line distance) • Empirical utilisation patterns were gathered by onsite survey of service users and used to adjust the boundaries between adjacent health facilities based on a ‘fuzzy choice’ algorithm	Statistical comparison of estimated catchment areas and empirical utilisation patterns, including direct comparison of boundaries produced by Thiessen polygons and the ‘fuzzy choice’ adjustment	Mean polygon boundary positions following adjustment; distance decay curves for indicator
Noor et al. [15]	Kenya	Subnational	Methods development and/or evaluation	Under 5 s	Fever treatment seeking	173 government hospitals, health centres and dispensaries	ArcView	• Travel time/impedance modelled using road network, land cover and digital elevation data assuming walking to the nearest health facility • Empirical utilisation patterns were gathered by onsite survey of service users and used to adjust the boundaries between adjacent health facilities based on a transect algorithm	Statistical comparison against the results produced by other catchment estimation methods: (i) travel time model without transect adjustment; (ii) straight-line distance to the nearest health facility	Map of one study district is presented to separately visualise catchment area boundaries and travel time strata produced by each model
Feikin et al. [61]	Kenya	Subnational	Estimation of catchment areas and/or population denominators	Under 5 s	Outpatient attendance	7 first level government or community health facilities	ArcView	• Denominators are enumerated population totals within polygons created by straight-line distance from geolocated residences to their nearest health facility	Statistical comparison of empirical utilisation patterns to denominators produced based on straight-line distance	Target population denominator for each named health facility; distance decay curve for indicator
Okiro et al. [59]	Kenya	Subnational	Other	Under 15 s	Malaria/all-cause admissions	17 district general hospitals	ArcGIS	• Admitted patients’ village of residence at defined timepoints were extracted from HMIS • Hospital catchment areas were defined algorithmically by selecting a set of enumeration areas representing 90% of admissions, then adjusted to smooth their boundaries and capture unassigned enumeration areas • Catchment denominators were estimated at defined timepoints using projected census enumeration area counts	None	Map visualising the locations of hospitals and their estimated catchment areas; baseline and projected target population denominator for each hospital
O’Meara et al. [66]	Kenya	Subnational	Model covariate computation	Under 5 s	Malaria-related admissions	10 public and 1 mission primary healthcare facilities	Not stated	• Travel time was modelled using road network data with nominal speeds for network walking time to the nearest health facility • Catchment areas were defined by assigning all children in the study area to their nearest health facility	None	Map visualising travel time to the nearest health facility; distance decay curves for indicator
Moïsi et al. [68]	Kenya	Subnational	Model covariate computation	Under 5 s; some stratified analyses in < 1 year and 1–4 years subgroups	Child mortality	5 hospitals and 47 vaccine clinics	ArcGIS	• Travel time/impedance modelled using road network, bus route and topographic data • Locations placed within specified travel time strata of the nearest health facility	Sensitivity analysis comparing multiple travel scenarios with nominal speeds: walking or walking/public transport, the latter assuming compound journey sequences	Maps separately visualising travel time to the hospital/nearest health facility by travel scenario
Okiro et al. [58]	Kenya	Subnational	Other	Under 15 s	Malaria/all-cause admissions	8 district general hospitals	ArcGIS	• Admitted patients’ village of residence at defined timepoints were extracted from HMIS • Hospital catchment areas were defined algorithmically by selecting a set of enumeration areas representing 90% of admissions, then adjusted to smooth their boundaries and capture unassigned enumeration areas • Catchment denominators were estimated at defined timepoints using projected census enumeration area counts	None	Projected target population denominator for each hospital
Moïsi et al. [69]	Kenya	Single health facility	Model covariate computation	Under 5 s	Child mortality	1 district hospital	ArcGIS	• Travel time/impedance modelled using road network, bus route and topographic data • Locations placed within specified travel time strata of the nearest health facility	Sensitivity analysis comparing multiple travel scenarios with nominal speeds: walking or walking/public transport, the latter assuming compound journey sequences	Maps separately visualising travel time to hospital by transport scenario; distance decay curve for indicator
Alegana et al. [18]	Namibia	Subnational	Estimation of catchment areas and/or population denominators	Under 5 s	Fever treatment seeking	All public health facilities within the study area	AccessMod; ArcGIS	• Travel time/impedance modelled using road network, land cover and digital elevation data • Travel time model assumes compound journey sequences to reach the nearest health facility • Travel time used as main predictor in a three-parameter logistic regression model to estimate treatment seeking probability • Threshold used to define catchment area extents was set based on review of the decay curve • Catchment denominators estimated by intersecting boundaries with population surface	Statistical comparison against the results produced by estimating straight-line distance to nearest health facility	Map visualising probability of treatment seeking by health facility with overlaid catchment area boundaries; distance decay curve for indicator; target population denominators aggregated by region and travel time strata
Blanford et al. [43]	Niger	National	Estimation of catchment areas and/or population denominators	Children aged 12–59 months	Complete vaccination status prior to 12 months of age	50 hospitals, 400 integrated health centres and 54 maternity centres	ArcGIS	• Travel time/impedance modelled using road network, land cover, surface water and digital elevation data • Travel times greater than 4 h to the nearest health facility taken as inadequate access • Kernel density estimation used with settlement population data to explore health service accessibility in areas with the high/low population	Sensitivity analysis comparing multiple travel scenarios with nominal speeds. Separate scenarios created for each combination of walking/vehicular travel and wet/dry season	Maps separately visualising (i) travel time to nearest health facility for each travel scenario, (ii) settlements by travel time for each season, highlighting those with no access
Kadobera et al. [65]	Tanzania	Subnational	Model covariate computation	Under 5 s; some stratified analyses in < 1 year and 1–4 years subgroups	Child mortality	Public health facilities; 13 dispensaries, 2 health centres and 2 district hospitals	ArcView	• Road network and footpath data used to estimate the path distance from each geolocated household to its nearest health facility along the shortest route	Statistical comparison against the results produced by estimating straight-line distance from each geolocated household to its nearest health facility	None presented
Mathiu et al. [37]	Multi-country study (The Gambia, Senegal)	Subnational	Estimation of catchment areas and/or population denominators	Under 5 s	Suspected meningitis incidence	Sentinel hospitals participating in the World Health Organisation meningitis network	OpenEpi	• District of residence retrieved from hospital records for each suspected meningitis case • Districts rank-ordered by number of cases contributed to the cumulative total • Catchment areas defined as the subset of rank-ordered districts contributing 80% of all cases • Hospital denominator estimated using district-level population	None	Target population denominator for each hospital
Manongi et al. [56]	Tanzania	Single health facility	Model covariate computation	Children aged 2–59 months	Hospital inpatient admissions and deaths	1 district hospital	ArcGIS; ArcView/AccessMod	• Straight-line distance between villages and the hospital were calculated • Catchment area was defined as the group of villages closer to the study hospital than any other • Catchment denominator population was extracted from national census data	None	Distance decay curves for indicators; target population denominators aggregated by travel time strata
Zaman et al. [63]	The Gambia	Subnational	Model covariate computation	Children aged 6–51 weeks at trial entry	Mortality, pneumonia incidence and pneumococcal vaccine efficacy	The two largest health facilities in the study area: one hospital and one health centre	ArcGIS	• Straight-line distance from compound of residence to the nearer of two study health facilities was calculated • Distance was categorised using a series of cut points	Statistical comparison against travel times derived from an isotropic time surface analysis	None presented
McLaren et al. [48]	South Africa	National	Model covariate computation	Separate subgroup analyses: over 18 s; under 5 s	Over 18 s: health consultation in the previous year; under 5 s: skilled attendant at birth	Public health facilities of all types	Not stated	• Straight-line distance from each geolocated household to its nearest health facility was calculated	None	Density curves for distance to the nearest health facility by population characteristics
World Health Organisation [39]	n/a	Single health facility	Estimation of catchment areas and/or population denominators	Under 5 s	Suspected meningitis incidence	Sentinel hospitals participating in the World Health Organisation meningitis network	n/a	• Hospital records to be reviewed for each suspected meningitis case (standard case definition) and district of residence retrieved • Districts rank-ordered by number of cases contributed to the cumulative total • Catchment area defined as the subset of rank-ordered districts contributing 80% of all cases • Hospital denominator estimated using district-level population	n/a	n/a
Aoun et al. [55]	Rwanda	Subnational	Model covariate computation	Under 5 s	Height-for-age z-score	All health centres and district hospitals in the study area	AccessMod/ArcGIS	• Travel time/impedance modelled using road network and land cover data • Catchment areas were defined in line with two constraints: (i) 1 h walking time to the nearest health facility, (ii) capacity of that facility	None	Maps separately visualising the location of health facilities, population density, land cover, travel time to the nearest health facility, and estimated catchment area boundaries
Afagbedzi [38]	Ghana	Subnational	Methods development and/or evaluation	All ages; subgroup under 5 s	Malaria and diarrhoea incidence (separately)	All health facilities within the study area	ArcGIS	• Catchment areas estimated by creating Thiessen polygons (straight-line distance) • Catchment denominators were estimated using the point-in-polygon method to aggregate the population of all census enumeration areas whose centroid fell within the catchment area	None	None presented
Macharia et al. [70]	Kenya	Subnational	Other	Pregnant women and infants	Uptake/allocation of long-lasting insecticidal bed nets	888 health facilities distributing long-lasting insecticidal bed nets and operated by government, non-governmental or faith-based institutions	ArcView; AccessMod; ArcGIS; R (including ‘geoR’ package)	• Travel time/impedance modelled using road network, land cover and digital elevation data • Travel time model assumes compound journey sequences to reach the nearest health facility • Result used as the main predictor in logistic regression models estimating the probability of health facility attendance • Additional model covariates were sourced from household survey data and converted to surface layers using ordinary kriging spatial interpolation • Threshold used to define catchment area extents was set based on review of the decay curve • Catchment denominators estimated by intersecting boundaries with population surface	Statistical comparison with independent data from the study area	Maps separately visualising travel time to nearest health facility and delineated catchment/unserved areas
Haidari et al. [45]	Mozambique	National	Estimation of catchment areas and/or population denominators	Girls aged 10 years	Access to immunisation centres	1337 routine immunisation centres for the World Health Organisation Expanded Program on Immunisation in Mozambique	SIGMA, a study-specific GIS platform	• Theoretical catchment areas defined by overlaying straight-line distance radii onto immunisation centre point locations • Catchment denominators estimated by intersecting boundaries with population surface • Population of the target demographic subgroup was approximated as a proportion of the total population figure	Sensitivity analysis to directly compare the results produced using several straight-line distance radii	Maps visualising the spatial coverage of progressively increasing catchment area radii
Smith et al. [49]	Uganda	National	Model covariate computation	Under 18 s; some stratified analyses in under 5 s and over 5 s subgroups	Unmet surgical need	All public health centres, general hospitals, district hospitals and regional referral hospitals with surgical capability	ArcGIS; QGIS; GeoDa	• Catchment areas estimated using a Voronoi diagram (straight-line distance) and the point-in-polygon method to encapsulate all enumeration areas centroids within each polygon	None	Maps visualising study outcomes and spatial associations in the indicator for surgical health facilities, aggregated to district level
Ouma et al. [19]	Kenya	Subnational	Model covariate computation	Under 5 s	Fever treatment seeking	All health facilities operated by government, faith-based and other non-profit organisations	ArcGIS; R (including ‘geoR’ package)	• Travel time/impedance modelled using road network, land cover and digital elevation data with nominal speeds • Travel time model assumes compound journey sequences to reach the nearest health facility • Result used as the main predictor in generalised linear mixed models to estimate treatment-seeking probability • Threshold used to define catchment area extents was set based on review of the decay curve • Catchment denominators estimated by intersecting boundaries with population surface	Candidate models were evaluated using a validation subset and accuracy metrics including classification error and receiver operating characteristic	Maps visualising separately visualising travel time to nearest health facility and probability of treatment seeking; distance decay curves for model covariates
Juran et al. [41]	Sub-Saharan Africa	Regional	Estimation of catchment areas and/or population denominators	All ages; subgroup under 15 s	Access to major hospitals providing surgical care	All major regional and district hospitals operated by local government, non-governmental or faith-based institutions	AccessMod	• Travel time/impedance modelled using road network, land cover and digital elevation data • Catchment areas estimated using 30 min, 1 h and 2 h travel time strata under two travel scenarios: walking on land cover and motorised transport on the road network • Catchment denominators estimated by intersecting boundaries with population surface adjusted for rate of surgical burden	None	Maps visualising total and percent population within travel time strata
Alegana et al. [40]	Sub-Saharan Africa	Regional	Model covariate computation	Under 5 s	Fever treatment-seeking	All major regional/district hospitals, dispensaries, clinics, health posts and health centres operated by government, local authority, faith based and non-governmental organisations	AccessMod; JAGS; R and rjags package	• Travel time/impedance modelled using road network, land cover and digital elevation data • Travel time model assumes compound journey sequences to reach the nearest health facility • Result used as the main predictor in a Bayesian model based on Item Response Theory to estimate treatment-seeking probability	Fitted model was evaluated using a validation subset and accuracy metrics including classification error and receiver operating characteristic	Maps separately visualising travel time to nearest hospital, health centre, and lower tier health facilities; distance decay curves for indicator
Kundrick et al. [47]	Malawi	National	Estimation of catchment areas and/or population denominators	Infants	Measles vaccination coverage; susceptible birth cohort; effective reproductive ratio	Unclear	Not stated	• Catchment areas estimated using a Voronoi diagram (straight-line distance) • Catchment denominators estimated by intersecting boundaries with population surface • Susceptible birth cohort denominator was approximated as 50% of the total population figure multiplied by the regional fertility rate	None	Maps separately visualising scaled measles vaccination coverage, susceptible birth cohort and effective reproductive ratio at health facility polygon level
Milucky et al. [57]	Burkina Faso	Single health facility	Estimation of catchment areas and/or population denominators	All ages; subgroups include children aged under 1 year, 1–2 years, 2–5 years, 5–15 years	Acute respiratory infection admission	1 district hospital	SAS, ArcGIS	• Commune of residence retrieved from hospital records for each admission • Communes rank-ordered by number of admissions contributed to the cumulative total • Catchment area defined as the subset of rank-ordered communes contributing 85% of all admissions • Hospital denominator estimated using commune-level population projections from census	None	Map visualising the location of communes comprising the hospital catchment area
Hierink et al. [78]	Mozambique	Subnational	Other	Under 5 s	Access to nearest health facility pre-/post-cyclone	All health facilities within the study area	QGIS; R (spatial packages not stated); AccessMod	• Travel time/impedance modelled using road network, land cover, surface water and digital elevation data • Travel time model assumes compound journey sequences to reach the nearest health facility • Accessibility defined using a maximum travel time of 2 h to the nearest health facility • Travel time extent intersected with population surface to estimate the number with access pre-/post-cyclone	Sensitivity analysis comparing travel scenarios based on upper/lower limits of motorised transport speed, and assumption that some floods waters are passable with reduced walking speed	Maps separately visualising areas with access to health facilities pre-/post-cyclone, and accessibility ratios at each timepoint
Joseph et al. [46]	Kenya	National	Model covariate computation	Children aged 12–23 months	DPT3 vaccination status; full immunisation status (BCG, measles, DPT3, polio and pneumococcal vaccines)	All public and private health facilities offering immunisation services	ArcMap; AccessMod	• Travel time/impedance modelled using road network, land cover and digital elevation data • Locations placed within specified travel time strata of the nearest health facility	Sensitivity analysis comparing multiple travel scenarios with nominal speeds: walking or walking/second transport mode (varying by terrain and road type/infrastructure), the latter assuming compound journey sequences	Maps separately visualising travel time to the nearest health facility by transport scenario
Alegana et al. [71]	Kenya	Subnational	Methods development and/or evaluation	Children aged 1 month to 14 years	Malaria/severe malaria admission	4 major level 4 or level 5 hospitals	ArcGIS; AccessMod; R (including ‘R-INLA’ package)	• Admitted patients’ enumeration area of residence were extracted from HMIS • Enumeration area-level probability of admission was modelled using a Bayesian hierarchical zero-inflated Poisson regression model • Catchment areas comprised of enumeration areas for which Bayesian posterior probability estimate exceeded a specified threshold	Candidate models were evaluated using a validation subset and metrics including cross-validated mean logarithmic score and root mean square error	Maps separately visualising the spatial distribution of admissions by enumeration area and conversion to hospital catchment areas
Mpimbaza et al. [60]	Uganda	Subnational	Estimation of catchment areas and/or population denominators	Children aged 1 month to 14 years	Malaria admissions	5 public district hospitals	Stata; R (for statistical analysis)	• Admitted patients’ parish of residence were extracted from HMIS • Catchment areas defined as the rural/peri-urban parishes contributing admissions that were located nearest to the hospital • Catchment denominators estimated using parish-level population projections from census	None	Map visualising the location of hospitals and their catchment areas; target population denominators expressed in population years at risk
Cairo et al. [50]	Democratic Republic of the Congo	Subnational	Estimation of catchment areas and/or population denominators	Children requiring paediatric surgical services	Access to paediatric surgical services	The highest level hospital or primary referral centre in each health district (40 in total); most were operated by public or faith-based organisations, but one private sector and one non-governmental organisation	AccessMod	• Accessibility defined using a maximum travel time of 2 h to the health facility, itself measured using 15 km straight-line distance as a proxy	None	Maps separately visualising the proportion of the target population with access to health facilities of different types, aggregated to district level
Epstein et al. [72]	Uganda	Subnational	Methods development and/or evaluation	Children between 6 months and 10 years of age	Malaria incidence	Two malaria surveillance centres	R (including ‘malariaAtlas’ package)	• Catchment areas were defined as those villages nearer to the specified health facility than any other • Travel time/impedance modelled using road network, land cover, surface water and digital elevation data • Travel time model assumes compound journey sequences to reach the nearest health facility • The mean travel time over all locations within the village was calculated, and used to quantify the effect of distance decay on the village-level probability of health facility utilisation • Catchment area denominators were estimated by ‘downweighting’ their projected population totals in line with distance decay, modelled using service utilisation data extracted from the HMIS	Statistical comparison of population-level incidence rates calculated using HMIS data and the estimated catchment denominators against independent data collected from a separate cohort study in the same area and population	Maps visualising the location of villages comprising health facility catchment areas and the probability of attendance by village; distance decay curves for probability of attendance
Cotache-Condor et al. [44]	Somaliland	National	Estimation of catchment areas and/or population denominators	Under 15 s	Access to surgical care	15 hospitals capable of providing surgical care	ArcMap	• Road network distance from households to the nearest hospital were converted to travel time assuming nominal and constant travel speed • The optimal catchment area extent was defined using a maximum travel time of 2 h, but locations were also placed within specified travel time strata of the hospital • Catchment denominators estimated by intersecting boundaries with population surface	Sensitivity analysis comparing multiple travel scenarios: walking or public transport	Maps visualising catchment areas for each hospital type by travel scenario and travel time strata
Simkovich et al. [42]	Multi-country study (Rwanda, Peru, India, Guatemala)	Subnational	Estimation of catchment areas and/or population denominators	Paediatric patients	Incidence of severe pneumonia	All public and private hospitals, health centres, health posts or other facilities in the study area treating paediatric patients	ArcGIS Pro, R (for statistical analysis and visualisation)	• Travel time/impedance modelled using road network and speed limit data assuming compound journey sequences • Locations placed within specified travel time strata of the nearest health facility • Strata boundaries intersected with population surface to estimate the proportion within each	Sensitivity analysis comparing multiple accessibility scenarios based on varying resource-/capability-based health facility characterisations	Maps separately visualising travel time to the nearest health facilities of different types
Hyde et al. [64]	Madagascar	Subnational	Methods development and/or evaluation	All ages; subgroup under 5 s	Malaria incidence	19 public health centres	QGIS/QuickOSM plugin; R (including ‘gstat’, ‘spdep’, ‘sp’ packages)	• Catchment areas defined by matching administrative units to their nearest health facility by the shortest average path distance over all households therein • Catchment denominators were compiled using administrative unit-level population estimates from the Ministry of Health • Population of the target demographic subgroup was approximated as a proportion of the total population figure	Additional data extracted from health facilities’ HMIS were used to estimate service utilisation and malaria incidence rates, and these were compared to independent cohort data from the same area to evaluate these estimates in terms of biases introduced by geographical/financial barriers	Maps visualising malaria incidence at specified timepoints by Fokontany

**Table 4:** Characteristics of the included publications

Characteristic	Number (%) of publications ^a^ (n = 35)
Study aims/product	
Methods development and/or evaluation	7 (20.0)
Estimation of catchment areas and/or population denominators	12 (34.3)
Computation of distance- or travel time-based model covariate	12 (34.3)
Other study aim(s)	4 (11.4)
Type of catchment estimation method or accessibility measure used	
Alignment with administrative boundaries	6 (17.1)
Straight-line distance	11 (31.4)
Network distance	3 (8.6)
Travel time/cost impedance modelling	11 (31.4)
Model-based geostatistics	4 (11.4)
Type of denominator estimation method used	
Aggregation of administrative unit population counts	11 (31.4)
Intersection of catchment boundaries with fine spatial-scale gridded population surface	12 (34.3)
Complete enumeration within Health and Demographic Surveillance System area	1 (2.9)
No denominator estimation	11 (31.4)
Data types used in the estimation process	
Purposive collection of origin/destination data	2 (5.7)
Data collected by Health and Demographic Surveillance System	5 (14.3)
Data extracted from Health Management Information System	7 (20.0)
Nationally representative household survey data	7 (20.0)
Spatial datasets, including road/footpath networks, land cover, topographic barriers to movement	22 (62.9)
Population counts by administrative unit	12 (34.3)
Fine spatial-scale gridded population surface	12 (34.3)
Source of data for health facility geolocation	
Onsite geolocation by field survey	5 (14.3)
Routine geolocation of all health facilities within Health and Demographic Surveillance System area	5 (14.3)
Study conducted in single or small number of health facilities	9 (25.7)
Data held by national health system	12 (34.3)
Regional database of geolocated public health facilities	2 (5.7)
Other open data source	2 (5.7)
Approach to evaluation of estimation methods	
Comparison against independent or purposively collected data	4 (11.4)
Direct comparison of multiple estimation methods using single analysis dataset	4 (11.4)
Sensitivity analysis or other statistical comparisons using single analysis dataset	10 (28.6)
Evaluation of candidate model specifications using a validation subset	3 (8.6)
No methods evaluation	14 (40.0)
Relevant estimation outputs presented	
Map delineating health facility catchment areas and boundaries	13 (37.1)
Isochrone map visualising health facility/service accessibility by travel time/distance strata	10 (28.6)
Distance decay curve for the health indicator of interest	10 (28.6)
Population denominators at level of individual health facilities	7 (20.0)
Population denominators at other level of spatial aggregation	6 (17.1)

^a^Table includes 33 peer-reviewed publications and 2 (of 3) grey literature reports (1 technical guidance document has been excluded)

### Study aims

While only 7 (20.0%) publications were framed around methods development and/or evaluation (Table 4), 12 (34.3%) each utilised similar methods to estimate catchment areas and/or population denominators, or to compute measures of health service accessibility as covariate to models testing associations with outcomes such as stunting [55] or hospital admission [56]. Several different health indicators were analysed (Table 3): indicators related to malaria or fever treatment-seeking were used for 15 (42.9%) publications, but 4 (11.4%) each analysed child mortality, access to/utilisation of surgical services, and immunisation coverage.

### Catchment area estimation

Catchment boundaries were sometimes (17.1%) aligned with those of established administrative units (Table 4), usually selected following an algorithmic process informed by patient-level data extracted from the HMIS [37, 57–60]. To produce catchment areas independent of administrative boundaries, many (31.4%) publications described the use of GIS software to assign locations to HFs based on straight-line distance [38, 47–52, 56, 61–63]. In cases where the network of roads and/or footpaths was also mapped, this was enhanced by measuring ‘network’ distance [64–66]. Similarly, additional spatial datasets describing other topographic features, such as land cover or slope, were often (31.4%) combined as input to cost impedance models measuring the travel time ‘cost’ of health-seekers’ most efficient route between locations [15, 19, 41–44, 46, 55, 67–69]. More complex approaches integrated spatial data with that gathered from nationally representative household surveys [18, 40, 70] or the HMIS [71] in geostatistical models assembling catchments based on location-specific estimates of health-seeking probability. Most publications presented a map of HF locations (65.7%) (Table 4) but, while those describing methods development or catchment estimation typically delineated their boundaries explicitly, others computing an accessibility-based model covariate tended to produce isochrones visualising travel time or distance strata.

### Denominator estimation

Although not all publications (68.6%) translated catchments to population denominators (Table 4), the methods for doing so were divided almost equally between intersecting catchment boundaries with fine spatial-scale gridded population surfaces (34.3%) and aggregating nationally- or locally-produced population counts (31.4%) at the level of districts [37], enumeration areas [38, 52], or villages [72], for example. Overall, 7 (20.0%) publications reported denominators for individual HFs and 6 (17.1%) at other levels of spatial aggregation.

### Evaluation of methods

Most (60.0%) publications described methods evaluation (Table 4), which commonly entailed sensitivity analyses (28.6%) or comparing multiple methods within a single analysis dataset (11.4%), but in the case of model-based approaches used a validation subset to assess the performance of candidate model specifications (8.6%). Only 4 (11.4%) described comparisons against independent [64, 70, 72] or purposively collected data [52].

### Data sources and software

Although most publications utilised open, or widely available, secondary data sources only (Table 4), several accessed data linking service utilisation events with health-seekers’ origin location, which are not routinely available in this setting: 5 (14.3%) were conducted within a Health and Demographic Surveillance System area [61, 65, 66, 68, 69], 7 (20.0%) extracted patient-level data from the HMIS [37, 57–60, 71, 72] and 2 (5.7%) surveyed health-seekers attending local HFs [15, 51].

Over half were published from 2017 onwards (Table 3). This may be linked to wider adoption of open-source software: while Geographic Information System (GIS) products such as ArcGIS and ArcView (Esri, Redlands, USA) and the AccessMod extension [73] were most common overall, use of R packages for spatial analysis (including ‘geoR’, ‘gstat’ and ‘R-INLA’) was evident from 2017 [19, 64, 71, 72].

## Discussion

This review of the literature on HF catchment population estimation for child health indicators in SSA found that few of the 36 included publications took methods development and/or evaluation as the primary focus. Of these, 7 were subnational research studies [15, 38, 51, 52, 64, 71, 72] and the eighth was a technical guidance document concerning single-hospital denominator estimation [39]. Though data inequity has previously been cited as a barrier [15], recent efforts to strengthen health data infrastructure and ongoing advances in the availability, coverage and resolution of spatial and demographic data may now offer the opportunity for development of reproducible methods that can be scaled to national-level networks. This will be essential if the HF is to be taken forward as a credible subnational unit for routine monitoring of health indicators.

**Fig. 2:**
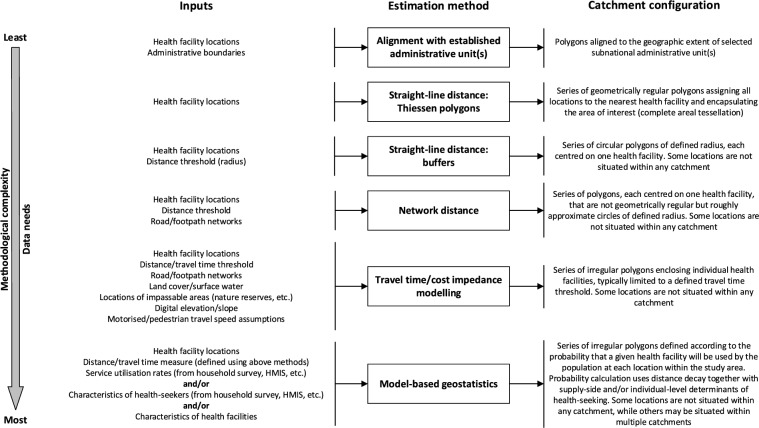
Outline of the catchment estimation methods identified by the review, together with their associated inputs and outputs

A successful catchment estimation method should, without need for empirical data tracing actual health-seeking flows, be able to outline the geographic area from which the users of a given HF are expected to originate [20]. The most basic method aligns catchments with established administrative units. This is problematic, however, in that subnational administrative boundaries do not usually impede population movement and are thus unlikely to accurately represent health-seeking flows. Most publications described methods underpinned by measures of spatial accessibility, which focus upon the space or distance separating health-seekers from services [74]. The chosen measure has implications for catchments’ extent, shape and configuration, however. By conceptualising the catchment network as a complete areal tessellation encapsulating the entire population [38, 47, 49, 51, 52], the simplest straight-line distance methods carry the unrealistic assumption that all health-seekers have access to one, and only one, HF. Recognising that some may, in reality, reside beyond practical reach of any HF, buffers were sometimes used to constrain catchments to a distance threshold provided by policy targets [46, 52, 55], guidance around the health indicator under consideration [41, 44, 50], or the inflection point of a modelled decay curve [18, 19, 70]. As straight-line distance inherently overlooks transport infrastructure and topographic barriers to movement, additional spatial data may be used to produce a more realistic measure [75]. Network distance may have limited utility in SSA, where pedestrian travel is common, and not necessarily restricted to roads and footpaths [76]. Instead, the process of converting the study area to a grid representation, assigning all cells a traversal ‘cost’ based on their aggregate spatial characteristics, then fitting cost impedance models to measure the travel time associated with health-seekers’ most efficient route to the nearest HF is often preferred, despite increased data and computational needs [75]. Aligning catchment boundaries more closely with topographical features that bar or facilitate movement may better represent the real-world travel experience in this setting, but is sensitive to the quality and resolution of spatial data. One publication noted that the coarse resolution required to handle the regional inconsistency of road network and other spatial data may have overestimated accessibility in rural areas or near major roads [41]. Distance measures or cost impedance models were sometimes integrated within a broader geostatistical modelling framework alongside other supply-side or individual-level factors, such that catchments were defined by the combined effect of multiple covariates on location-specific probability of health-seeking and service utilisation [70, 71]. Overall, the included publications depicted a trade-off between catchment estimation methods that are comparatively easy to implement, but oversimplistic and likely to yield unrealistic denominators, and others that more accurately represent reality but entail additional data needs and methodological complexity (Fig. 2).

Increasingly complex modelling approaches present the further challenge that the optimal specification and appropriateness of any assumptions are likely context-specific [75, 77], underlining the need for evaluation. While cost impedance modelling facilitated comparison of alternate travel scenarios based on variable transport modes [44, 69] or seasonal conditions [43, 78] as a means of sensitivity analysis, geostatistical approaches further allowed for the performance of candidate model specifications to be compared using a validation dataset [19, 40, 71]. Nonetheless, empirical ‘origin/destination’ data specifying health-seekers’ origin location and the HF attended, though scarce in SSA, expose actual health-seeking flows and should be seen as the ‘gold standard’ for evaluation. Where available, these data provided new, otherwise unattainable insight: two related studies collecting origin/destination data via onsite surveys showed that travel beyond the nearest HF was common [15, 51]. Catchment boundaries bisecting the space between adjacent HFs typify straight-line distance methods, but the finding that a substantial proportion of health-seekers attended a more distant, but higher-tier, HF (hospitals rather than health centres, for example) suggests that inter-facility competition or other HF characteristics may also influence health-seeking and, as such, have a role in appropriate boundary placement. Indeed, while accessibility measures, in isolation, implicitly assume that all health-seekers attend, and can be served by, the nearest HF, methods adjusting for facilities’ capacity [55] or capabilities in respect of specific conditions [42], or health-seekers’ individual-level characteristics [19, 70], for example, may produce more realistic results. Though simpler methods based on spatial accessibility have arguably been necessary in the absence of data specifying health-seekers’ origin location or capturing the range of aspatial supply-side and individual-level factors known to influence patient choice [20], recent advances may now permit the use of more comprehensive, yet scalable, methods leveraging multiple data sources with national coverage.

Directly linking estimation methods to RCHD could help to narrow the gap between modelling and reality. Two publications [37, 57] followed disease-specific technical guidance issued by the World Health Organisation [39, 79], which proposed algorithmic case detection and geolocation from retrospective hospital records then catchment delineation at the geographic extent of rank-ordered administrative units contributing a cumulative 80–85% of cases. Though a relatively simple and intuitive algorithm, replication is limited by the burden of manual retrieval and review of physical records, which were often difficult to locate, incomplete or illegible [57]. Instead, clinical surveillance databases appear a more practicable foundation for algorithm development [60] or amalgamation of RCHD from multiple HFs [71, 72]. Although the included publications described local, purpose-built databases, they lend credence to the notion that HMIS, bolstered by recent strengthening initiatives, may be a viable platform for scalable estimation methods. Indeed, DHIS2 has been instrumental to the development of a novel approach to district-level denominator and intervention coverage estimation [80], subsequently replicated elsewhere in SSA [81, 82]. Having thus far been applied in established administrative units only, this method did not meet the review inclusion criteria but may have potential at more granular geographies such as HF catchments. Perhaps reflecting the long-standing prominence of malaria within the international health agenda [83], nearly half of the included publications focussed on related indicators, with other pressing concerns such as lower respiratory infections and diarrhoeal disease [84, 85] comparatively underrepresented. The breadth of RCHD could address this imbalance by enabling parallel, indicator-specific estimation using a common methodological approach, a valuable innovation given the propensity for health-seeking and distance decay to vary by type or severity of health event [61, 86]. Realising these aspirations will depend on consistent, complete and high-quality data throughout the health system, however, a concern that has historically led to structural underutilisation of RCHD in SSA [87]. Although embedding standard data entry procedures and automated quality assessment tools within electronic HMIS may alleviate some quality issues, continued efforts to strengthen the manual, paper-based data capture processes and tools used by health workers should remain a priority [21, 24]. Few studies captured both public and private HFs, which has rarely been possible in SSA owing to suboptimal reporting by the private sector [88, 89]. There is a need for additional policy measures targeted to eliminating this gap so that the entire HF network can be factored into routine monitoring and decision-making processes.

**Fig. 3:**
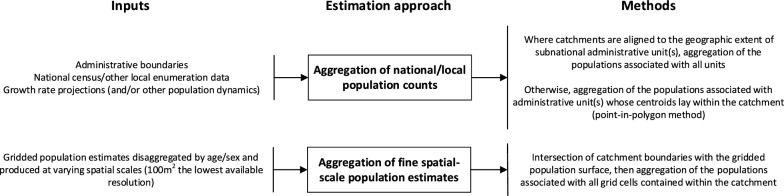
Outline of the inputs and methods for denominator estimation identified by the review

Imprecision was evident in translating catchment areas to population denominators. Most publications followed one of two broad approaches (Fig. 3). The first, applied where catchments were aligned with established administrative units, estimated denominators by aggregating nationally- or locally-produced population counts. These counts were typically projected using objective growth rates, and, in one case, were ‘downweighted’ in line with distance decay [72]. Recent advances in the production of spatially disaggregated demographic data have enabled an alternate, GIS-based approach intersecting catchment boundaries with fine spatial-scale gridded population surfaces. Though this may improve the precision of denominators associated with non-standard administrative/spatial units, such as catchments [13], the gains may be attenuated if small-area population demographics are unknown, necessitating subgroup approximation as a proportion of the total cell count [47]. Similar advances in temporal granularity are also needed; reliance on temporally coarse data, such as the decennial census, has meant that catchment denominators are effectively treated as static counts despite fluctuating in response to short-term population movement [90], individual travel behaviours [91], seasonal conditions [43, 78] and disease epidemiology [57]. Aggregated mobile phone call records have shown promise for tracking spatio-temporal population dynamics [90–92] and could contextualise longitudinal service utilisation patterns discerned from RCHD, speaking to the potential of hybrid methods drawing upon multiple data sources. Further methodological enhancement would be needed, however, to address the selection biases associated with HF utilisation [87] and mobile phone ownership [93].

Having taken HF catchment areas as the spatial unit of interest, this review has a distinct focus to much of the research literature on small-area and subnational population estimation and contributes to the fields of public health and spatial demography. The breadth of the review was a strength, having employed a search strategy bridging geospatial, epidemiological and demographic methods for the estimation of HF catchment populations, and gathering peer-reviewed and grey literature from several sources. It is acknowledged, however, that relevant publications utilising such methods may have been omitted if substantive methodological content was absent from titles or abstracts. Moreover, by limiting the review to the health sector methods unique to education, or other public services [20], may have been excluded.

## Conclusion

This review found that most studies implemented estimation methods using data from a single or subset of HFs only. Such methods are unlikely to be generalisable if benefitting from well-developed and robust data infrastructure unrepresentative of the wider health system, underlining the need for investment in methods that can be scaled to national-level HF networks. Whilst considerable methodological variation was observed, standardised and scalable methods could be achieved by leveraging data sources that are readily available at national scale, such as RCHD, nationally representative household surveys and spatially disaggregated demographic data. Many publications focussed on indicators related to malaria, but RCHD could also help to fulfil the need for population denominators in respect of other heath conditions. Although quality concerns have historically resulted in underutilisation of RCHD in SSA, emphasising their value for catchment population estimation could accelerate quality improvement initiatives and efforts to improve private sector reporting rates. Future methodological development should move away from using accessibility measures in isolation towards geostatistical approaches uniting spatial characteristics of health service supply with the broader range of supply-side, individual-level and environmental factors that may exert an influence on health-seekers’ choice behaviour. In particular, explicitly accounting for inter-facility competition in catchment estimation could help to overcome the commonplace, but likely invalid, assumption of attendance to the nearest facility. Future research should also consider the potential of adapting innovative approaches utilised in other sectors, disciplines or high-income countries for HF catchment population estimation in SSA.

## Supplementary Information

Supplementary material 1.

Supplementary material 2.

## Data Availability

All of the research publications and grey literature reports included in this review have been cited, and full description of the search strategy is presented in the online supplementary information. Other review materials are available from the corresponding author on reasonable request.

## Abbreviations

DHIS2: District health information software
GIS: Geographic information systems
HMIS: Health management information system
LMIC: Low- and middle-income country
PRISMA-ScR: Preferred reporting items for systematic reviews and meta-analyses extension for scoping reviews
RCHD: Routinely collected health data
SSA: Sub-Saharan Africa
SDGs: Sustainable development goals
