# Supplementary material for: A scoping review of the methods used to estimate health facility catchment populations for child health indicators in sub-Saharan Africa
Source: Popul Health Metr. 2025 Mar 29;23:11. doi: 10.1186/s12963-025-00374-0 (PMC11955140; doi:10.1186/s12963-025-00374-0)
Supplement: Supplementary file 1 — Supplementary material 1. [file 12963_2025_374_MOESM1_ESM.docx]

**Supplementary file 2: Review search strings for bibliographic databases**

The review search string was initially developed using the Medline bibliographic database, and consisted of MeSH terms and search terms arranged into four broad ‘concepts’ (representing children, health facilities, catchment areas/population denominators and sub-Saharan Africa) using Boolean operators. Thereafter, and following peer review by two research librarians at the University of Southampton, the completed Medline search string was translated to the Scopus, Web of Science Core Collection, GeoBase and African Index Medicus databases. This entailed the removal of MeSH terms, and other database-specific syntax amendments, as appropriate.

To identify relevant grey literature, the same search string was further translated to the EBSCO platform, as a general source of grey literature, and the ProQuest database, for dissertations and theses. Accordingly, the ‘Dissertations & Theses’ filter was applied when searching the ProQuest database.

The final strings used to search each of the above-mentioned databases are presented below.

**Medline:**

(neonat*.mp. OR newborn*.mp. OR bab*.mp. OR infan*.mp. OR child.mp. OR children.mp. OR "under five*".mp. OR "under 5*".mp. OR infant/ OR Child, Preschool/)

AND (health facilit*.mp. OR health center*.mp. OR health centre*.mp. OR health service*.mp. OR hospital*.mp. OR Health Facilities/)

AND (denominator*.mp. OR catchment*.mp. OR Catchment Area, Health/

OR (

(access*.mp. OR Health Services Accessibility/)

AND (geograph*.mp. OR GIS.mp. OR geospatial.mp. OR spatial.mp. OR Geographic Information Systems/ OR Spatial Analysis/)

)

)

AND (

(“sub”.mp. AND sahar*.mp. AND africa*.mp.) OR “Africa South of the Sahara"/ OR angola.mp. OR benin.mp. OR botswana.mp. OR “burkina faso”.mp. OR burundi.mp. OR “cabo verde”.mp. OR “cape verde”.mp. OR cameroon.mp. OR “central african republic”.mp. OR chad.mp. OR comoros.mp. OR congo.mp. OR “c?te d’ivoire”.mp. OR “ivory coast”.mp. OR djibouti.mp. OR eritrea.mp. OR ethiopia.mp. OR gabon.mp. OR gambia.mp. OR ghana.mp. OR guinea.mp. OR kenya.mp. OR lesotho.mp. OR liberia.mp. OR madagascar.mp. OR malawi.mp. OR mali.mp. OR mauritania.mp. OR mauritius.mp. OR mayotte.mp. OR mozambique.mp. OR namibia.mp. OR niger.mp. OR nigeria.mp. OR r?union.mp. OR rwanda.mp. OR “s?o tom? and princip?”.mp. OR senegal.mp. OR seychelles.mp. OR “sierra leone”.mp. OR somalia.mp. OR “south africa”.mp. OR “south sudan”.mp. OR sudan.mp. OR swaziland.mp. OR eswatini.mp. OR tanzania.mp. OR togo.mp. OR uganda.mp. OR zambia.mp. OR zimbabwe.mp.

)

**Scopus:**

( TITLE-ABS-KEY ( neonat* OR newborn* OR bab* OR infan* OR child OR children OR "under five*" OR "under 5*" ) )

AND ( TITLE-ABS-KEY ( "health facilit*" OR "health center*" OR "health centre*" OR "health service*" OR hospital* ) )

AND ( ( TITLE-ABS-KEY ( denominator* OR catchment* ) )

OR (

( TITLE-ABS-KEY ( access* ) )

AND ( TITLE-ABS-KEY ( geograph* OR "GIS" OR geospatial OR spatial ) )

 )

 )

AND (

( TITLE-ABS-KEY ( sub AND sahar* AND africa* ) ) OR ( TITLE-ABS-KEY ( angola OR benin OR botswana OR "burkina faso" OR burundi OR "cabo verde" OR "cape verde" OR cameroon OR "central african republic" OR chad OR comoros OR congo OR "c?te d'ivoire" OR "ivory coast" OR djibouti OR eritrea OR ethiopia OR gabon OR gambia OR ghana OR guinea OR kenya OR lesotho OR liberia OR madagascar OR malawi OR mali OR mauritania OR mauritius OR mayotte OR mozambique OR namibia OR niger OR nigeria OR r?union OR rwanda OR "s?o tom? and princip?" OR senegal OR seychelles OR "sierra leone" OR somalia OR "south africa" OR "south sudan" OR sudan OR swaziland OR eswatini OR tanzania OR togo OR uganda OR zambia OR zimbabwe ) )

)

**Web of Science Core Collection:**

TS=(neonat* OR newborn* OR bab* OR infan* OR child OR children OR "under five*" OR "under 5*")

AND TS=("health facilit*" OR "health center*" OR "health centre*" OR "health service*" OR hospital)

AND TS=(denominator* OR catchment*

OR (

access*

AND (geograph* OR GIS OR geospatial OR spatial)

)

)

AND TS=((sub AND sahar* AND africa*) OR angola OR benin OR botswana OR “burkina faso” OR burundi OR “cabo verde” OR “cape verde” OR cameroon OR “central african republic” OR chad OR comoros OR congo OR “c?te d’ivoire” OR “ivory coast” OR djibouti OR eritrea OR ethiopia OR gabon OR gambia OR ghana OR guinea OR kenya OR lesotho OR liberia OR madagascar OR malawi OR mali OR mauritania OR mauritius OR mayotte OR mozambique OR namibia OR niger OR nigeria OR r?union OR rwanda OR “s?o tom? and princip?” OR senegal OR seychelles OR “sierra leone” OR somalia OR “south africa” OR “south sudan” OR sudan OR swaziland OR eswatini OR tanzania OR togo OR uganda OR zambia OR zimbabwe)

**GeoBase:**

((neonat* WN KY) OR (newborn* WN KY) OR (bab* WN KY) OR (infan* WN KY) OR (child WN KY) OR (children WN KY) OR ("under five*" WN KY) OR ("under 5*" WN KY))

AND (({health facility} WN KY) OR ({health facilities} WN KY) OR ({health center} WN KY) OR ({health centers} WN KY) OR ({health centre} WN KY) OR ({health centres} WN KY) OR ({health service} WN KY) OR ({health services} WN KY) OR (hospital* WN KY))

AND ((denominator* WN KY) OR (catchment* WN KY)

OR (

(access* WN KY)

AND ((geograph* WN KY) OR (GIS WN KY) OR (geospatial WN KY) OR (spatial WN KY))

)

)

AND (((sub WN KY) AND (sahar* WN KY) AND (africa* WN KY)) OR (angola WN KY) OR (benin WN KY) OR (botswana WN KY) OR ({burkina faso} WN KY) OR (burundi WN KY) OR ({cabo verde} WN KY) OR ({cape verde} WN KY) OR (cameroon WN KY) OR ({central african republic} WN KY) OR (chad WN KY) OR (comoros WN KY) OR (congo WN KY) OR ({ivory coast} WN KY) OR ((c?te WN KY) AND (d'ivoire WN KY)) OR (djibouti WN KY) OR (eritrea WN KY) OR (ethiopia WN KY) OR (gabon WN KY) OR (gambia WN KY) OR (ghana WN KY) OR (guinea WN KY) OR (kenya WN KY) OR (lesotho WN KY) OR (liberia WN KY) OR (madagascar WN KY) OR (malawi WN KY) OR (mali WN KY) OR (mauritania WN KY) OR ( mauritius WN KY) OR (mayotte WN KY) OR (mozambique WN KY) OR (namibia WN KY) OR (niger WN KY) OR (nigeria WN KY) OR (r?union WN KY) OR (rwanda WN KY) OR ((s?o WN KY) AND (tom? WN KY) AND (princip? WN KY)) OR (senegal WN KY) OR (seychelles WN KY) OR ( {sierra leone} WN KY) OR (somalia WN KY) OR ({south africa} WN KY) OR ({south sudan} WN KY) OR (sudan WN KY) OR (swaziland WN KY) OR (eswatini WN KY) OR (tanzania WN KY) OR (togo WN KY) OR (uganda WN KY) OR (zambia WN KY) OR (zimbabwe WN KY))

**African Index Medicus:**

(tw:(neonat* OR newborn* OR bab* OR infan* OR child OR children OR "under five*" OR "under 5*"))

AND (tw:("health facility" OR "health facilities" OR "health center" OR "health centers" OR "health centre" OR "health centres" OR "health service" OR "health services" OR hospital*))

AND ((tw:(denominator* OR catchment*))

OR (

(tw:(access*))

AND (tw:(geograph* OR GIS OR geospatial OR spatial))

)

)

AND ((tw:((sub AND sahar* AND africa*))) OR (tw:(angola)) OR (tw:(benin)) OR (tw:(botswana)) OR (tw:("burkina faso")) OR (tw:(burundi)) OR (tw:(“cabo verde”)) OR (tw:(“cape verde”)) OR (tw:(cameroon)) OR (tw:(“central african republic”)) OR (tw:(chad)) OR (tw:(comoros)) OR (tw:(congo)) OR (tw:((co?e AND d'ivoire))) OR (tw:(“ivory coast”)) OR (tw:(djibouti)) OR (tw:(eritrea)) OR (tw:(ethiopia)) OR (tw:(gabon)) OR (tw:(gambia)) OR (tw:(ghana)) OR (tw:(guinea)) OR (tw:(kenya)) OR (tw:(lesotho)) OR (tw:(liberia)) OR (tw:(madagascar)) OR (tw:(malawi)) OR (tw:(mali)) OR (tw:(mauritania)) OR (tw:(mauritius)) OR (tw:(mayotte)) OR (tw:(mozambique)) OR (tw:(namibia)) OR (tw:(niger)) OR (tw:(nigeria)) OR (tw:(r?union)) OR (tw:(rwanda)) OR (tw:((s?o AND tom? AND princip?))) OR (tw:(senegal)) OR (tw:(seychelles)) OR (tw:(“sierra leone”)) OR (tw:(somalia)) OR (tw:(“south africa”)) OR (tw:(“south sudan”)) OR (tw:(sudan)) OR (tw:(swaziland)) OR (tw:(eswatini)) OR (tw:(tanzania)) OR (tw:(togo)) OR (tw:(uganda)) OR (tw:(zambia)) OR (tw:(zimbabwe)))

**ProQuest:**

(

noft(neonat* OR newborn* OR bab* OR infan* OR child OR children OR "under five*" OR "under 5*")

AND stype.exact("Dissertations & Theses")

)

AND (

noft("health facilit*" OR "health center*" OR "health centre*" OR "health service*" OR hospital*)

AND stype.exact("Dissertations & Theses")

)

AND (

noft(denominator* OR catchment*

OR (

access*

AND (geograph* OR GIS OR geospatial OR spatial)

))

AND stype.exact("Dissertations & Theses")

)

AND (

noft(("sub” AND sahar* AND africa*) OR angola OR benin OR botswana OR "burkina faso" OR burundi OR "cabo verde" OR "cape verde" OR cameroon OR "central african republic" OR chad OR comoros OR congo OR “c?te d’ivoire” OR “ivory coast” OR djibouti OR eritrea OR ethiopia OR gabon OR gambia OR ghana OR guinea OR kenya OR lesotho OR liberia OR madagascar OR malawi OR mali OR mauritania OR mauritius OR mayotte OR mozambique OR namibia OR niger OR nigeria OR “r?union” OR rwanda OR (s?o AND tom? AND princip?) OR senegal OR seychelles OR “sierra leone” OR somalia OR “south africa” OR “south sudan” OR sudan OR swaziland OR eswatini OR tanzania OR togo OR uganda OR zambia OR zimbabwe)

AND stype.exact("Dissertations & Theses")

)

**EBSCO:**

(

TI(neonat* OR newborn* OR bab* OR infan* OR child OR children OR "under five*" OR “under 5*”)

OR AB(neonat* OR newborn* OR bab* OR infan* OR child OR children OR "under five*" OR “under 5*”)

OR SU(infant OR Child, Preschool)

)

AND (

TI(“health facilit*” OR “health center*” OR “health centre*” OR “health service*” OR hospital*)

OR AB(“health facilit*” OR “health center*” OR “health centre*” OR “health service*” OR hospital*)

OR SU(Health Facilities)

)

AND (

(

TI (denominator OR catchment)

OR AB (denominator OR catchment)

OR SU (Catchment Area, Health)

)

OR (

(

TI (access*)

OR AB (access*)

OR SU (Health Services Accessibility)

)

AND (

TI (geograph* OR GIS OR geospatial OR spatial)

OR AB (geograph* OR GIS OR geospatial OR spatial)

OR SU (Geographic Information Systems OR Spatial Analysis)

)

)

)

OR (

TI(“sub” AND sahar* AND africa*)

OR AB(“sub” AND sahar* AND africa*)

OR SU(Africa South of the Sahara)

OR TI(angola OR benin OR botswana OR “burkina faso” OR burundi.mp. OR “cabo verde” OR “cape verde” OR cameroon OR “central african republic” OR chad OR comoros OR congo OR “c?te d’ivoire” OR “ivory coast” OR djibouti OR eritrea OR ethiopia OR gabon OR gambia OR ghana OR guinea OR kenya OR lesotho OR liberia OR madagascar OR malawi OR mali OR mauritania OR mauritius OR mayotte OR mozambique OR namibia OR niger OR nigeria OR r?union OR rwanda OR (sao AND tome AND principe) OR senegal OR seychelles OR “sierra leone” OR somalia OR “south africa” OR “south sudan” OR sudan OR swaziland OR eswatini OR tanzania OR togo OR uganda OR zambia OR zimbabwe)

OR AB(angola OR benin OR botswana OR “burkina faso” OR burundi.mp. OR “cabo verde” OR “cape verde” OR cameroon OR “central african republic” OR chad OR comoros OR congo OR “c?te d’ivoire” OR “ivory coast” OR djibouti OR eritrea OR ethiopia OR gabon OR gambia OR ghana OR guinea OR kenya OR lesotho OR liberia OR madagascar OR malawi OR mali OR mauritania OR mauritius OR mayotte OR mozambique OR namibia OR niger OR nigeria OR r?union OR rwanda OR (sao AND tome AND principe) OR senegal OR seychelles OR “sierra leone” OR somalia OR “south africa” OR “south sudan” OR sudan OR swaziland OR eswatini OR tanzania OR togo OR uganda OR zambia OR zimbabwe)

)
