# Supplementary material for: A scoping review of the methods used to estimate health facility catchment populations for child health indicators in sub-Saharan Africa
Source: Popul Health Metr. 2025 Mar 29;23:11. doi: 10.1186/s12963-025-00374-0 (PMC11955140; doi:10.1186/s12963-025-00374-0)
Supplement: Supplementary file 2 — Supplementary material 2. [file 12963_2025_374_MOESM2_ESM.docx]

**Supplementary file 3: Searches of relevant organisational websites**

A simplified search strategy was used to search Google Scholar and relevant organisational websites. This consisted of targeted keyword combinations representing the broad search ‘concepts’ that had already been used in the search strings used to identify research publications from bibliographic databases. However, the children concept was not explicitly included in these searches in favour of identification by screening the search results, and the sub-Saharan Africa concept was not applied to allow for methodological guidance that was not linked to specific countries to be screened and included in the review, where appropriate.

As a simple area-based headcount is a practicable method that may be employed by health facility workers in sub-Saharan Africa to estimate the population of service users, the synonymous terms “head count” and “headcount” were taken as additional terms that may represent the catchment area ‘concept’ in some settings. The possibility that this term may be more prevalent in grey rather than research literature, was tested by searching the Medline database using an adapted search string that replaced the catchment area ‘concept’ with the “head count” and ”headcount” search terms together. This search returned only 4 results, each of which was found to be inappropriate for the scoping review. As such, the “head count” and ”headcount” search terms were excluded from the bibliographic database search strategy.

Thus, separate searches were run on each organisational website, and using each unique combination of the following terms (using one from each ‘concept’):

- Health facilities: “health facility”, “health center”, “hospital”
- Catchment areas/population denominators: “denominator”, “catchment”, “head count”/”headcount”

The following websites were searched:

| **Date of search** | **Organisation** | **Website URL** |
| --- | --- | --- |
| 31^st^ January 2022 | Population Council | <https://www.popcouncil.org/research/results?resources=yes&projects=yes&experts=yes> |
| 31^st^ January 2022 | World Health Organisation | <https://www.who.int/home/search> |
| 31^st^ January 2022 | UNICEF | <https://www.unicef.org/search> |
| 31^st^ January 2022 | World Bank | <https://www.worldbank.org/en/search> |
| 31^st^ January 2022 | USAID | <https://www.usaid.gov/site-search/> |
| 1^st^ February 2022 | Demographic and Health Survey | <https://dhsprogram.com/publications/Publication-Search.cfm> |
| 1^st^ February 2022 | Countdown to 2030 Collaboration | <https://www.countdown2030.org/publications> |
| 1^st^ February 2022 | Institute for Health Metrics and Evaluation | <https://www.healthdata.org/search> |
| 1^st^ February 2022 | MEASURE Evaluation | <https://www.measureevaluation.org/publications.html> |
| 2^nd^ February 2022 | ICF International | <https://www.icf.com/search> |
| 2^nd^ February – 4^th^ February 2022 | Google Scholar | <https://scholar.google.com/> |
| 20^th^ June 2022 | DHIS2 | <https://dhis2.org/> |
